# Unveiling the Impact of Drying Methods on Phytochemical Composition and Antioxidant Activity of *Anthemis palestina*

**DOI:** 10.3390/plants12223914

**Published:** 2023-11-20

**Authors:** Mahmoud A. Al-Qudah, Hala I. Al-Jaber, Faten M. Abu Orabi, Hazem S. Hasan, Amal K. Aldahoun, Abdulrahman G. Alhamzani, Abbas I. Alakhras, Tareq T. Bataineh, Abdel Monem M. Rawashdeh, Sultan T. Abu-Orabi

**Affiliations:** 1 Department of Chemistry, College of Science, Imam Mohammad Ibn Saud Islamic University (IMSIU), Riyadh 11623, Saudi Arabia; agalhamzani@imamu.edu.sa (A.G.A.); aakhrasi@imamu.edu.sa (A.I.A.); 2 Department of Chemistry, Faculty of Science, Yarmouk University, P.O. Box 566, Irbid 21163, Jordan; 2019104001@ses.yu.edu.jo (A.K.A.); tariq.b@yu.edu.jo (T.T.B.); rawash@yu.edu.jo (A.M.M.R.); abuorabi@yu.edu.jo (S.T.A.-O.); 3 Department of Chemistry, Faculty of Science, Al-Balqa Applied University, Al-Salt 19117, Jordan; hala.aljaber@bau.edu.jo; 4 Department of Chemistry, Faculty of arts and Science, Applied Science Private University, Amman 11937, Jordan; f_aladwan@asu.edu.jo; 5 Department of Plant Production and Protection, Faculty of Agricultural Technology, Al-Balqa Applied University, Al-Salt 19117, Jordan; hazem@bau.edu.jo; 6 Department of Medical Analysis, Faculty of Science, Tishk international University, Erbil 44001, KRG, Iraq

**Keywords:** *Anthemis palestina*, essential oil composition, impact of drying methods, TPC, TFC, antioxidant activity, LC-MS/MS

## Abstract

Different drying techniques may alter the chemical composition of plant extracts and consequently affect their bioactivity potential. The current study was designed to reveal the effect of four different drying methods on the phytochemical composition and antioxidant activity of hydrodistilled essential oil (HD-EO) and methanolic (APM) extract obtained from the aerial part of *Anthemis palestina* from Jordan. Aerial parts of *A. palestina* in their fresh (FR) form and after drying in shade (ShD), sun (SD), oven at 40 °C (O40D) and 60 °C (O60D), in addition to microwave (MWD), were used to extract their essential oils by hydrodistillation and to prepare the different methanolic extracts (APM). GC/MS analysis of the different HD-EOs revealed qualitative and quantitative differences among the different samples. While FR, O40D, O60D, and MWD EO samples contained mainly sesquiterpene hydrocarbons (35.43%, 29.04%, 53.69%, and 59.38%, respectively), ShD sample was rich in oxygenated monoterpenes (33.57%), and SD-EO contained mainly oxygenated sesquiterpenes (40.36%). Principal component analysis (PCA) and Cluster analysis (CA) grouped the different drying methods based on their impact on the concentration of chemical constituents. SD-EO demonstrated high DPPH and ABTS antioxidant activity (1.31 ± 0.03) × 10^−2^; (1.66 ± 0.06) × 10^−2^ μg/mL, respectively). Furthermore, *A. paleistina* methanolic extracts (APM) obtained after subjecting the plant to different drying methods showed interesting patterns in terms of their TPC, TFC, antioxidant activity, and phytochemical profiling. Of all extracts, SD-APM extract had the highest TPC (105.37 ± 0.19 mg GA/g DE), highest TFC (305.16 ± 3.93 mg Q/g DE) and demonstrated the highest DPPH and ABTS scavenging activities ((4.42 ± 0.02) × 10^−2^; (3.87 ± 0.02) × 10^−2^ mg/mL, respectively); all were supported by correlation studies. LC-MS/MS analysis of the different extracts revealed the richness of the SD-APM extract in phenolic acids and flavonoids.

## 1. Introduction

Essential oils (EOs) are complex natural combinations of volatile molecules that are usually extracted from the plant material through hydrodistillation and are well recognized for their wide spectrum of bioactivities. EOs and their constituents have been classified as antioxidants [1,2,3,4,5], food preservatives [6,7,8], and insect repellents [9,10,11] in the past. Exogenous parameters such as soil characteristics, harvest time, plant growth stage, geographical location, extraction technique, and/or drying method were all found to have a significant impact on the extractive yield, chemistry, and related bioactivities [12].

Due to the short lifetime of most medicinal herbs, fresh plant material is seldom used for a long time during the year. Herbal remedies are usually collected and then preserved by drying for prolonged use during the whole year. While drying can preserve plant material by preventing enzymatic spoilage, it can alter the yield and chemical composition of EOs and extracts, including constituents like polyphenols, pigments, and vitamins [13]. Different drying methods may have pronounced effects on the chemical composition of EOs/extracts [14,15,16], and thus their bioactivity might change. According to the literature, the most common method employed for plant drying is drying in oven [14,17,18,19,20]. Accordingly, preservation of medicinal plants requires the employment of the optimal drying method to preserve and enhance the content and bioactivity.

*Anthemis* is one of the largest genera of the Asteraceae (Compositeae) family that comprises approximately 210 species [21]. Plants belonging to this genus are known for their wild geographical distribution including Europe, Southwestern Asia, Northern and Northeastern Africa, Southern Arabia, and tropical East Africa [22,23]. Here in Jordan, the *Anthemis* genus is represented by 16 species only [24]. *Anthemis* species are reputed in folk medicine in many cultures for their anti-inflammatory, antioxidant, antibacterial, and antispasmodic effects [25,26]. Different classes of secondary metabolites were isolated from several *Anthemis* plants including sesquiterpene lactones [27], polyacetylenes [28,29,30,31], and flavonoids [32]. *Anthemis* species assayed previously for their essential oil composition [33,34] contained β-pinene, α-pinene, spathulenol, germacrene D, caryophyllene oxide, and limonene [33,34,35,36,37,38,39] as main constituents.

The current study was designed to investigate the effect of different drying methods (natural ones (drying in shade (ShD); Sun drying (SD)) and artificial (oven drying at two different temperatures (40 °C: O40D); 60 °C (O60D); microwave drying (MWD))) on the volatile (essential oil) and nonvolatile chemistry of *Anthemis palestina*, from Jordan. The impact of these different drying methods was assessed in terms of their effect on the chemical constituents, total phenol content (TPC), total flavonoid content (TFC), and antioxidant activity, determined by the DPPH and ABTS methods, in addition to their effect on the methanolic extract composition.

## 2. Results and Discussion

### 2.1. Composition of HDEO Obtained from the Plant Material Subjected to Different Drying Methods

**Figure 1:**
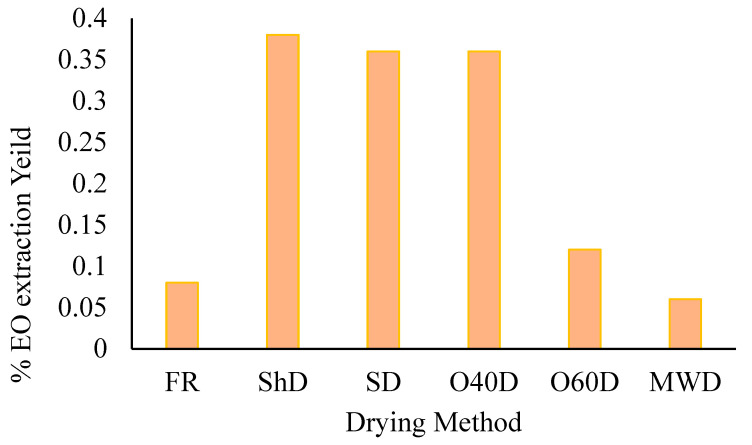
*A. palestina* EO yields variation in essential oil extraction yield (*w*/*w*) with the employed drying method (FR: Fresh; ShD: Shade-dried; SD: Sun-dried; O40D: Oven-dried at 40 °C; O60D: Oven-dried at 60 °C; MWD: Microwaved-dried).

Hydrodistillation of the aerial parts of *A. palestina* afforded essential oils at varying yields (Figure 1), with the highest yield obtained from the plant material subjected to ShD (0.38%, by weight) and the lowest resulting from microwave (100 W)-dried plant material (0.06% by weight).

### 2.2. GC/MS Analysis of HD-EOs Obtained from A. palestina Aerial Parts Subjected to Different Drying Methods

Each HD-EO obtained from the plant material after being subjected to the selected drying method was analyzed by GC/MS technique to reveal its chemical constituents; the results are listed in Table 1 (Figure S1). The analysis resulted in the identification of 165 constituents with notable qualitative and quantitative variations. In total, 102, 72, 78, 84, 81, and 75 components were identified in the HD-EOs, representing 95.21%, 97.74%, 96.83%, 97.36%, 66.69%, and 99.09% of the total content in FR, ShD, SD, O40D, O60D, and MWD samples, respectively. The identified compounds were grouped into six main groups based on their chemical structures. These were aliphatic compounds (AC), carboxylic acids and esters (CE), monoterpene hydrocarbons (MH), oxygenate monoterpenes (OM), sesquiterpene hydrocarbons (SH), and oxygenate sesquiterpenes (OS).

The HD-EO obtained from FR plant sample was dominated by SH and OS, which amounted to 35.43% and 30.82% of the total composition, respectively. Monoterpenes and their oxygenated derivatives made an appreciable contribution to the total composition as well, with the OM being detected at higher concentration levels as compared to MH (16.31%, 7.54%, respectively). The main components detected in this FR-EO sample were γ-muurolene (11.20%), terpinen-4-ol (5.47%), (E)-caryophyllene (3.51%), (E)-β-farnesene (3.20%), sylvestrene (3.19%), and α-zingiberene (3.10%). In the HD-EO obtained from plant samples dried in shade (ShD), OM were the main contributors, amounting to 33.57% of the total composition followed by SH (26.61%). This variation in composition was attributed to the detection of higher concentration levels of terpinen-4-ol (16.94%) as compared to its content in the FR-EO sample. This variation in concentration levels was also observed for γ-muurolene (6.82%), α-zingiberene (5.46%), and (Z)-β-farnesene (5.11%).

The SD-HD-EO was characterized by high content of sesquiterpenes and their oxygenated derivatives, both accounting in total for 69.29% of the total composition. The content of OM detected in this sample was almost similar to the one obtained from FR-EO (16.74%). The main individual representatives of SD-EO included terpinen-4-ol (9.35%), spathulenol (7.96%), α-cedrene epoxide (6.65%), γ-muurolene (6.00%), and (Z)-β-farnesene (5.91%).

The HD-EOs obtained from O40D, O60D, and MWD were characterized by high content of SH (29.04%; 53.69%; and 59.38%, respectively). Again, qualitative and quantitative variations among the individual constituents were observed. The main constituents detected in the O40D-EO included each of terpinen-4-ol (12.91%), γ-muurolene (7.95%), and (E)-caryophyllene (5.42%). The main constituents in O60D-EO were γ-muurolene (18.69%), (E)-β-farnesene (10.92%), α-zingiberene (8.60%), and caryophyllene oxide (6.60%). It was observed that the main constituents in the MWD-EO were like those detected in the O60D-EO, but with variable concentration levels. The main constituents in the MWD-EO were γ-muurolene (18.73%), α-zingiberene (14.70%), (Z)-β-farnesene (9.26%), and α-(E, E)-farnesene (5.12%).

In our current study, it was noticed that upon increasing the temperature during the drying process (specifically, oven drying at 40 °C and 60 °C), terpenoidal content varied. Drying at high temperature lowered the total content of monoterpenes but increased the total content of sesquiterpenes. This was in total agreement with the previous work [19,40,41], mainly being attributed to the higher volatility of monoterpenes as compared to sesquiterpenes.

### 2.3. Principal Component Analysis and Cluster Analysis

In the current study, principal component analysis (PCA) and cluster analysis (CA) were applied to the data shown in Table 1, in order to decipher the underlying patterns in the concentrations of the different constituents detected in the oils extracted from *A. palestina* aerial parts subjected to different drying methods.

**Figure 2:**
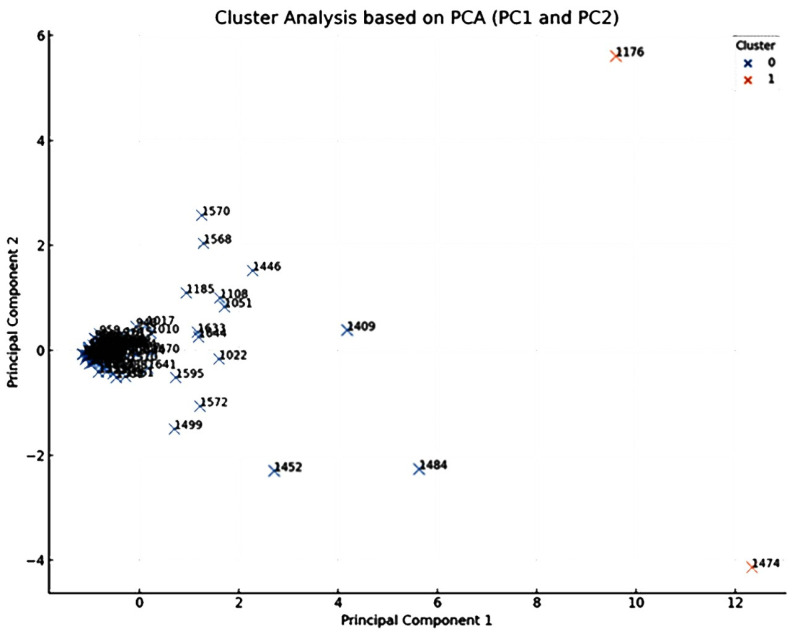
PCA loadings plot, based on the first two principal components PC1 and PC2 of the HDEOs constituents (identified by their Kovat Index KI). Each point represents a chemical constituent identified by its Kovats index, detected in the oils obtained from *A. palestina* subjected to different drying methods (*p* value > 0.05).

The obtained PCA scatter plot, showing the scores of individual chemical components on the primary two principal components (PC1 and PC2) is shown in Figure 2. Each point within this plot corresponds to a distinct chemical component, identified by its KI value as listed in Table 1. The distribution of these points encapsulates their concentration profiles across an array of the different drying techniques employed in the current study including the FR, ShD, SD, O40D, O60D, and MWD methods.

Close and careful inspection of this scatter plot revealed an interesting observation related to the emergence of two main clusters that signified chemical components with identical concentration profiles across the different drying techniques. Components located proximally on the plot share analogous profiles, signifying similar responses to the drying methods, while those distantly placed exhibit divergent concentration profiles, implying differential responses to the drying processes.

Components that are prominently positioned on the extremities of the plot, especially along PC1 and PC2 axes, require special attention. The strategic position of these components underscores their distinct concentration profiles, deviating markedly from the average response. These components, particularly γ-muurolene (1474), terpinen-4-ol (KI 1176), and α-zingiberene (1484), could be significant as potential biomarkers for this genus/species.

**Figure 3:**
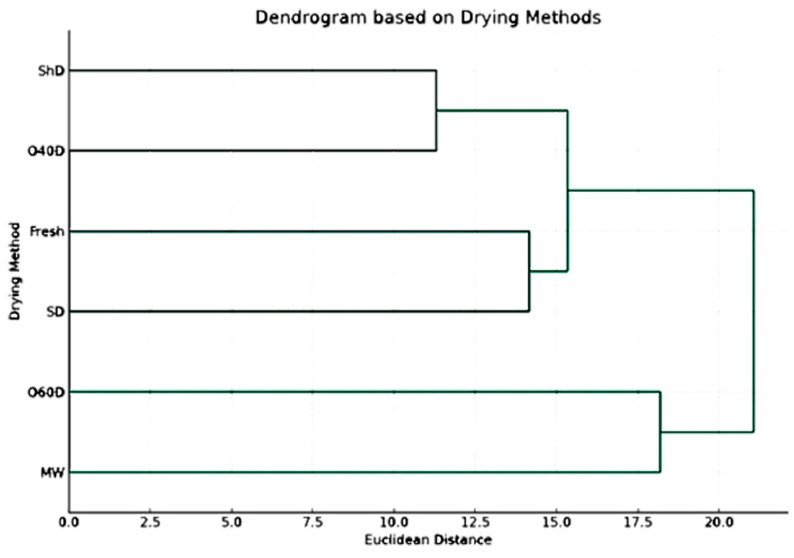
The dendrogram is based on drying methods, including untreated fresh plant material (*p* value > 0.05).

To confirm and better understand the obtained results, a dendrogram based on drying methods was established (Figure 3) in which the different drying methods are grouped based on their impact on the concentration of chemical components, revealing a hierarchy of which drying methods resulted in similar chemical profiles. Based on the results shown in this dendrogram, it is clear that the chemical profiles of the HD-EO obtained from the plant material subjected to drying in shade (ShD) and oven drying at 40 °C (O40D) were most similar as compared to other profiles resulting from other drying techniques. The second group of similar chemical profiles corresponded to FR and SD EO-samples.

Understanding the effect of drying methods on the EO composition and which could produce similar chemical profiles can be very helpful in taking the correct informative decisions during extraction and preservation processes.

**Figure 4:**
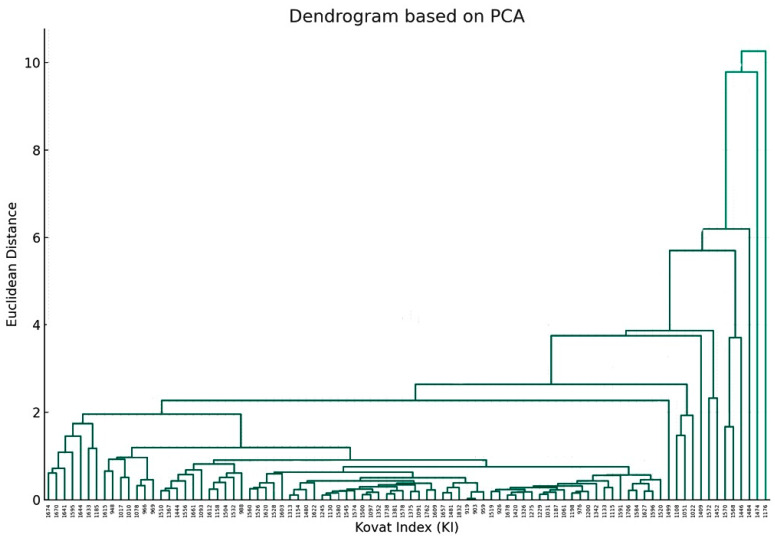
The dendrogram visualizes the hierarchical clustering of the chemical components based on their PCA results. The *x*-axis represents the Kovat Index (KI) for each chemical component (as mentioned in Table 1), and the *y*-axis represents the Euclidean distance, which indicates similarity (the closer the distance is to zero, the more similar the components are) (*p* value > 0.05).

Furthermore, another dendrogram, based on PCA, that focuses on the relationship between individual components was also obtained (Figure 4).

The fusion of branches at varying heights indicates the degree of similarity between components, with lower heights indicating greater similarity. Components that merge at shorter distances on the *y*-axis exhibited similar concentration profiles across drying methods. The farther up the *y*-axis these merges occur, the more dissimilar the components are.

Joining information obtained from the two dendrograms, one can recognize which drying method is optimal for enhancing or diminishing specific chemical components. If a particular cluster of chemical components (Figure 4) is considered beneficial, the drying method dendrogram (Figure 3) can guide towards the method that maximizes these components.

### 2.4. Antioxidant Activities of HD-EOs

In the present investigation, the antioxidant activities of HD-EOs extracted from the aerial parts of *A. palestina* subjected to different drying methods were determined and compared to the two positive controls, ascorbic acid and α-tocopherol (Table 2 and Figure S2).

The HD-EO obtained from FR plant material had the highest DPPH radical scavenging activity as compared to other HD-EOs (IC_50_: (1.00 ± 0.03) ×10^−2^ μg/mL). The EOs obtained from plant material subjected to SD and O40D were comparable ((1.31 ± 0.03) × 10^−2^; (1.66 ± 0.06) × 10^−2^ μg/mL, respectively). These two EOs had also the highest ABTS scavenging activity ((1.34 ± 0.01) × 10^−2^; (1.91 ± 0.01) × 10^−2^ μg/mL, respectively). Results in Table 2 clearly demonstrate the effect of drying method on the antioxidant activity. These findings are in total agreement not only with those listed in the literature [42] but also with the results of statistical analysis performed in our current investigation, that indicated the effect of the different drying methods on the chemical composition. Again, combining the information obtained from dendrograms in Figure 3 and Figure 4 together can offer guidance in choosing the proper drying method that maximizes the beneficial effect (in this case, the antioxidant activity).

### 2.5. Total Phenol Content (TPC), Total Flavonoid Content (TFC) and Antioxidant Activity of APM Extracts

The different *A. palestina* methanolic extracts (APM) extracts were tested for their TPC and TFC according to the procedure described in the literature [1,2,3,7]. The obtained results are listed in Table 2.

As could be deduced from the results obtained in Table 3, APM extract obtained from the SD plant material had the highest TPC (105.37 ± 0.19 mg GA/g DE) and TFC (305.16 ± 3.93 mg Q/g DE). The extract obtained from the plant material subjected to O60D had the lowest TPC and TFC (43.49 ± 0.57 mg GA/g DE, 52.94 ± 0.90 mg of Q/g DE, respectively).

The results demonstrated that extracts obtained from the plants dried at the low temperatures (ShD and SD) had the highest TPC when compared to those obtained at higher temperatures (O40D, O60D, and MWV), thus confirming the effect of drying methods on the composition (especially the phenolics content) [43]. The high phenolic content of the extract obtained from MW-dried plant material could be attributed to the effect of microwave radiation and its elevated temperature on the plant tissue, especially the cell wall. This drying method could have enabled the release of cell wall phenolics, and consequently increased their measured content [44].

Most extracts showed moderate to high TFC, except for the extract obtained from the plant dried in the oven at 60 ºC, which had the lowest content (52.94 ± 0.90 mg Q/g DE).

In our study, DPPH and ABTS radical scavenging methods were used to evaluate the antioxidant activity of the different APM extracts; the results are summarized in Table 3 and Figure S3. Most tested extracts had interesting scavenging activities, in the two methods, with the APM-SD extract having the highest DPPH and ABTS methods ((4.42 ± 0.02) × 10^−2^; (3.87 ± 0.02) × 10^−2^ mg/mL, respectively). The observed strong antioxidant activity of this extract could be correlated with its high TPC and TFC. The lowest DPPH and ABTS activities were recorded for APM-O60D (IC_50_ of (17.51 ± 1.72) × 10^−2^ and (23.99 ± 1.62) × 10^−2^ μg/mL, respectively) which was characterized with the lowest TPC and TFC. Our findings strongly support the effect of different drying methods on the observed antioxidant activity of plant extracts [45,46].

### 2.6. Correlation Studies: Antioxidant Activities, TPC, and TFC

In the current study, the obtained data (TPC, TFC, DPPH, and ABTS) were evaluated to calculate the correlation matrix of the different investigated variables (phenolic and flavonoid contents with antioxidant activities). The calculated correlation matrices are displayed in Table 4 (Figures S6 and S7).

As could be deduced from the obtained correlation results, there is a very strong correlation (0.99027) between TPC and TFC, suggesting that these two variables are positively related.

The data revealed also strong negative correlations observed between each of TPC and DPPH (−0.95541); TPC and ABTS (−0.95987); TFC and DPPH (−0.95703); and TFC and ABTS (−0.94990). These strong negative correlations indicate an inverse relationship between each pair, confirming the effect of higher phenolic and flavonoid contents on the observed DPPH and ABTS antioxidant activities; as the content increases, IC_50_ value decreases, indicating stronger antioxidant activity. There was also a positive correlation observed between the DPPH and ABTS (0.84878).

### 2.7. LC-MS Analysis of Phytochemicals

In the current investigation, the presence of a selected set of constituents in the APM extracts obtained from aerial parts of *A. palaestina,* dried as described previously, was determined by LC-MS/MS using both the positive and negative ionization modes. The total ion chromatograms (TICs) of the four APM extracts corresponding to the different drying methods employed in the current study are shown in Figure S4; results of the LC-Ms/MS analysis are summarized in Table 5. A total of 43 compounds were detected and identified, including 23 flavonoids, 5 organic acids, 6 phenolic acids, and 10 other compounds. Several compounds were common to all analyzed extracts and were mostly reported to occur in the plants belonging to the same family. The findings of this analysis are in total agreement with those of TPC and TFC, and support the high antioxidant activity observed for the extracts, especially for the APM-SD extract that contained most of the detected compounds, including all phenolic acids and flavonoids.

## 3. Materials and Methods

### 3.1. Plant Material

The aerial parts of *A. Palestina* were collected from Samou region in Irbid, Jordan, during the full flowering stage (the spring of 2022). The identity of the plant was confirmed using characteristics related to growth habits and morphological attributes in regional floras [24] and was further confirmed by Prof. Dr Jamil Al-Lahham, Department of Biological Sciences—Faculty of Science, Yarmouk University, Irbid, Jordan. A voucher specimen was deposited in the herbarium of the Department of Biological Sciences-Yarmouk University, Irbid, Jordan (YU/09/ AA/1002).

### 3.2. Drying Conditions

Fresh aerial parts of *A. Palestina* were subjected to different drying methods, immediately after collection. The different drying methods included drying in shade (ShD), sun drying (SD), oven drying at 40 °C (O40D), oven drying at 60 °C (O60D), and microwave drying (MWD). In the ShD method, the aerial parts of the plant (250 g) were dried in a dark dry room with appropriate ventilation until constant weight was achieved (4 weeks, at 25 ± 2 °C). In SD, the aerial parts (250 g) were dried on paper trays under direct sunlight at temperatures ranging between 19 °C and 36 °C for 7 days until constant weight was achieved. In the oven drying (OD) method, the aerial parts of the plant (250 g) were dried in a ventilated oven at two selected temperatures separately, 40 °C and 60 °C, for 3 days to assure constant weight achievement. In MWD (100 W), same amount of aerial plant material was dried for 3 min.

### 3.3. Extraction of Essential Oil

Each dried sample (250 g) was subjected to hydrodistillation for 4 h using a Clevenger-type apparatus. The obtained oils were collected, dried over anhydrous MgSO_4,_ and then stored in sealed amber vials and kept at 4 °C until analysis was performed.

### 3.4. Preparation of the Methanolic Extract

Air-dried (ShD) and grinded aerial parts of *A. palestina* were subjected to Soxhlet extraction with petroleum ether to get rid of fatty material and waxes. Then, the dried defatted plant residue was extracted in the same apparatus with methanol three times. The combined methanolic extract was evaporated under reduced pressure and the obtained residue (APM) was then assayed for its total phenol content (TPC), total flavonoid content (TFC), and antioxidant activity using DPPH and ABTS assay methods. The extract was then further assayed for its chemical constituents by HPLC-MS/MS analysis.

### 3.5. GC and GC-MS Analysis

A sample of 1 μL of each oil was diluted to 3.0 μL with GC grade *n*-hexane, and then analyzed by GC-MS (Chromatec Crystal GC-MSD, Yoshkar-Ola, Russia) equipped with a CR-5 MS column (5% diphenyl, 95% dimethyl polysiloxane, 30 m × 0.25 mm, 0.25-μm film thicknesses). In the MS detector, an electron ionization mode of 70 eV was used. The temperature in the MS source was set at 300 °C and transfer line temperature at 230 °C. The temperature column was programmed from 40 °C for 1 min (isothermal) to 280 °C at a constant rate of 3 °C/min, with the lower and upper temperatures being held constant for 3 min. Helium was used as a carrier gas (1.0 mL/min). The relative peak areas were used to calculate the relative percentage concentrations of the detected compounds. A standard solution of C_8_–C_30_ *n*-alkanes mixture was analyzed under the same chromatographic conditions. The chemical constituents of the essential oils were identified by comparing their calculated Kovats retention index (KI) (relative to *n*-alkanes C_8_–C_30_), matching their recorded mass spectra with the built-in library spectra and by comparing their mass spectra to those of authentic standards.

### 3.6. Total Phenolic and Total Flavonoid Contents

The total phenolic and total flavonoid contents (TPC, TFC, respectively) of the APM extracts obtained from each drying method were determined by two assay methods, including the Folin–Ciocalteu and aluminum chloride methods, respectively, as previously described [1,2].

### 3.7. Antioxidant Activity of HDEOs and Methanolic Extracts

The antioxidant activity of the different APM extracts and HDEOs was evaluated by the DPPH and ABTS methods as described in [1,2,3,7]. The scavenging ability was calculated based on the following equation:

% Activity = [(Ac − As)/Ac] × 100,

where Ac is the absorbance of the control, and As is the absorbance in the presence of extracts or standards.

### 3.8. LC-MS Analysis of Phytochemicals

Secondary metabolites profiling of the crude methanolic extract (APM) was done using a Bruker Daltonik Elute UHPLC system equipped with Impact II ESI-Q-TOF System (Bremen, Germany), in both the positive [M + H]^+^ and negative [M − H]^−^ electrospray ionization modes. Isothermal chromatographic separation was performed on a C-18 reversed phase column (100 × 2.1 mm, 1.8 µm, 120 Å, Bruker Daltonik, Germany) at 30 °C with a total elution time of 20 min. The autosampler temperature was kept constant at 8 °C. Plant samples were dissolved with 2.0 mL DMSO, and then the total volume of the sample was completed to 50 mL using acetonitrile (HPLC grade). The sample was centrifuged at 4000 rpm for 2 min before injection (injection volume was 3.0 µL). Secondary metabolite constituents in the studied extract were identified based on their *m*/*z* ratio with reference to the retention time of the used standards.

### 3.9. Statistical Analysis

Data analysis was carried out with SAS software version 9.4 (2013). TPC, TFC, DPPH, ABTS were analyzed by Pearson Correlation and one-way ANOVA model with Drying methods. Before ANOVA, descriptive statistics for all the measurements were made in order to observe the distribution of the data and check the normality by general linear model (*p* value > 0.05). Means were separated using LSD test at a significance level of 0.05.

## 4. Conclusions

The current study revealed the impact of employing different drying methods on the phytochemical composition and antioxidant activities of essential oils and methanolic extracts obtained from the aerial parts of *A. palestina* growing wild in Jordan. Indeed, the employed drying methods affected the chemical composition of the HF-EOs qualitatively and quantitatively. Principal components analysis and cluster analysis grouped the different drying methods according to their impact on the chemical composition. The EO obtained from the sun-dried plant material had the highest antioxidant activity due to its chemical profile. This was also in total agreement with chemical profiling and antioxidant activity tests observed for the different APM extracts. Again, the extract obtained from the SD plant material had the highest TPC and TFC, and accordingly demonstrated the highest antioxidant activity in the two assay methods. Correlation studies confirmed these findings and were all in agreement with the LC-MS/MS results, which indicated the richness of the SD-APM extract in phenolic compounds and flavonoids. Consequently, this HD-EO and APM extract obtained from SD method showed the highest favorable antioxidant activity. This study underscores the importance of recognizing the impact of drying methods on the chemical profiles of EOs and extracts. Such studies can help in taking the correct decision in employing the correct drying technique for obtaining the best-desired extract with optimal composition and activity. In the current investigation, of the different drying methods used, sun drying of plant material afforded methanolic extract with favorable composition and antioxidant activity.

## Figures and Tables

**Table 1:** Chemical compositions of HD-EO obtained from *A*. *palestina* aerial parts, subjected to different drying methods, in comparison to the fresh (FR) sample.

No.	KI	Constituent	% Concentration					
			FR	ShD	SD	O40D	O60D	MW
1	816	n-octane	-	-	-	-	-	0.40
2	841	(E)-2-Hexenal	0.25	0.15	-	-	-	-
3	846	(Z)-Salvene	0.20	0.09	-	-	-	-
4	857	Ethyl isovalerate	-	-	-	-	-	0.13
5	859	(Z)-2-Hexenol	0.72	-	-	-	-	-
6	862	n-Hexenol	0.35	-	-	-	-	-
7	901	Heptanal	-	0.20	-	-	-	0.11
8	903	2-Ethoxy ethyl acetate	-	0.60	0.81	-	-	0.35
9	919	2-methyl-4-Heptanone	-	0.63	0.77	-	-	0.33
10	926	Tricyclene	0.29	0.48	0.13	0.15	0.19	0.79
11	927	Artemisia triene	-	-	-	1.36	-	-
12	939	Allyl isovalerate	-	0.14	0.16	-	-	-
13	941	α-Pinene	-	-	-	0.08	-	-
14	948	Benzaldehyde	0.19	2.68	0.44	1.26	1.26	0.16
15	959	(E)-2-Heptenal	-	0.73	1.06	-	-	0.47
16	966	Sabinene	-	0.74	-	1.38	0.09	-
17	969	β-Pinene	0.18	0.54	-	2.12	0.17	0.28
18	973	*trans*-*m*-Mentha-2,8-diene	-	-	-	0.09	-	-
19	976	6-methyl-5-Hepten-2-one	-	0.25	-	0.14	0.17	-
20	983	3-ρ-Menthene	-	0.16	-	-	-	-
21	988	Myrcene	1.57	0.19	0.39	0.67	0.16	1.20
22	989	α-Phellandrene	-	-	-	0.31	-	-
23	999	δ-3-Carene	-	-	-	0.15	-	-
24	1010	α-Terpinene	0.45	1.85	0.29	3.09	0.99	0.49
25	1017	ρ-Cymene	0.37	2.49	0.39	2.30	0.98	0.29
26	1022	Sylvestrene	3.19	2.37	0.68	3.21	1.09	2.41
27	1031	Benzene acetaldehyde	0.25	-	-	0.14	0.17	-
28	1042	(E)-β-Ocimene	0.26	-	-	-	-	-
29	1051	γ-Terpinene	1.03	4.24	0.58	5.62	2.01	0.94
30	1052	Isobutyl acetoacetate	-	-	-	-	-	0.46
31	1061	*cis*-Sabinene hydrate	0.33	0.53	-	0.23	-	-
32	1063	*cis*-Linalool oxide	-	0.28	-	-	-	-
33	1078	Terpinolene	0.30	1.12	-	1.40	0.47	0.21
34	1091	Linalool	0.31	0.24	0.63	0.14	0.35	0.46
35	1093	*trans*-Sabinene hydrate	0.97	1.40	-	1.23	0.30	-
36	1097	n-Nonanal	0.32	-	0.50	-	-	0.58
37	1098	*cis*-Thujone	-	0.69	-	0.63	-	-
38	1104	2-Methyl butyl isovalerate	-	-	-	0.11	-	-
39	1108	*trans*-Thujone	2.46	5.02	0.46	4.11	0.44	0.37
40	1115	*cis*-ρ-Menth-2-en-1-ol	0.27	0.59	0.21	0.53	0.26	-
41	1130	*trans*-Pinocarveol	0.20	0.17	0.31	0.15	-	-
42	1133	(E)-Tagetone	0.47	0.9	-	0.78	0.46	-
43	1151	Camphor	-	-	-	0.10	-	-
44	1154	neo-menthol	0.41	-	1.17	-	-	0.40
45	1158	*cis*-Chrysanthenol	0.99	0.37	-	0.13	-	-
46	1176	Terpinen-4-ol	5.47	16.94	9.35	12.91	4.88	2.43
47	1178	ρ-Cymen-8-ol	-	0.21	-	-	-	-
48	1183	*trans*-ρ-Mentha-1(7),8-dien-2-ol	-	-	-	-	-	-
49	1184	Thuj-3-en-10-al	-	-	-	0.12	-	-
50	1185	α-Terpinenol	1.05	3.33	2.03	2.64	0.93	0.44
51	1187	Myrtenol	0.21	0.28	-	-	0.31	-
52	1189	*cis*-Piperitol	-	-	-	0.20	-	-
53	1198	n-Decanal	-	-	0.16	0.12	0.10	-
54	1200	*trans*-Piperitol	-	0.36	0.26	0.26	0.13	-
55	1204	*trans*-Pulegol	-	-	-	0.13	0.15	-
56	1209	Nerol	-	0.40	-	0.22	-	-
57	1214	*trans*-Chrysanthenyl acetate	-	-	-	0.09	0.13	-
58	1229	(Z)-3-hexenyl-3-methyl Butanoate	0.34	0.18	-	0.16	-	-
59	1236	Hexyl isovalerate	0.37	-	-	-	-	-
60	1245	Geraniol	0.26	-	0.29	0.14	0.16	-
61	1275	Isobornyl acetate	0.25	0.15	0.17	0.19	0.36	0.41
62	1288	3-Thujanol acetate	-	-	-	0.09	0.10	-
63	1307	(2E,4Z)-Decadienal	-	-	0.20	-	-	-
64	1313	Methyl geranate	0.32	-	1.24	0.10	-	0.24
65	1326	δ-Elemene	0.35	-	-	0.11	0.13	0.47
66	1342	dimethoxy-(E)-Citral	-	0.59	-	0.38	0.27	0.72
67	1344	1-Phenyl pentan-3-one	0.54	-	0.83	-	-	-
68	1348	α-Terpinylacetate	0.14	-	-	-	-	-
69	1352	Nerylacetate	0.15	-	0.34	-	-	0.34
70	1367	α-Ylangene	0.77	0.90	0.84	0.92	1.20	0.68
71	1372	Geranyl acetate	0.19	-	-	-	-	-
72	1375	α-Copaene	0.46	0.20	0.78	0.35	0.53	0.50
73	1380	iso-Longifolene	-	-	-	-	0.18	-
74	1381	7-epi-Sesquithujene	0.53	-	0.31	-	-	0.34
75	1385	(E)2-Octenol butanoate	0.31	-	0.29	-	-	-
76	1389	Methyl eugenol	-	-	-	0.13	-	-
77	1402	iso-Italicene	0.48	-	-	-	-	0.21
78	1409	(E)-Caryophyllene	3.51	3.89	4.37	5.42	5.63	3.87
79	1420	β-Copaene	0.42	0.23	-	0.19	0.31	0.39
80	1434	Iridolactone	0.50	-	-	-	-	-
81	1441	Aromadendrene	-	-	-	-	-	0.16
82	1444	α-Humulene	0.96	0.76	0.81	1.19	1.31	0.58
83	1446	(Z)-β-Farnesene	-	5.11	5.91	-	-	9.26
84	1449	epi-β-Santalene	-	0.17	0.24	-	-	-
85	1452	(E)-β-Farnesene	3.20	-	-	5.08	10.92	0.20
86	1464	*allo*-Aromadendrene	0.39	-	0.21	-	-	-
87	1474	γ-Muurolene	11.2	6.82	6.00	7.95	18.69	18.73
88	1476	γ-Gurjunene	-	0.23	2.59	-	-	-
89	1480	methyl-β-(E)-Ionol	-	-	-	0.12	-	-
90	1479	Amorpha-4,7(11)-diene	2.11	-	-	-	-	-
91	1480	Phenyl ethyl 2-methylbutanoate	0.63	-	1.04	-	-	0.53
92	1481	β-Selinene	0.22	-	1.10	0.22	0.16	-
93	1482	δ-Selinene	-	-	-	0.12	-	-
94	1484	α-Zingiberene	3.10	5.46	1.70	4.49	8.60	14.70
95	1490	Bicyclogermacrene	2.27	-	-	-	0.34	-
96	1497	Isodaucene	-	1.84	0.61	-	-	-
97	1498	α-Muurolene	0.26	-	-	-	0.16	-
98	1499	α-(E,E)-Farnesene	1.85	-	-	1.11	2.22	5.12
99	1500	β- Bisabolene	0.34	-	0.45	0.08	-	0.32
100	1502	β-Dihydro agarofuran	-	-	0.79	-	-	-
101	1504	δ-Amorphene	1.11	0.25	-	0.36	0.61	0.84
102	1510	δ-Cadinene	0.72	0.75	0.60	0.81	1.43	0.85
103	1519	(E)-dihydro-Apofarnesal	0.41	0.44	-	-	0.21	0.46
104	1520	β-Sesquiphellandrene	0.36	-	0.56	0.39	1.26	0.25
105	1526	(E)-iso-γ-Bisabolene	0.82	-	1.58	0.12	-	1.00
106	1528	Raspberry ketone	0.29	0.33	1.53	0.50	0.38	0.70
107	1530	α-Calacorene	-	-	-	-	0.14	-
108	1532	Hedycaryol	1.08	-	0.42	-	1.19	0.94
109	1535	Italicene epoxide	-	-	-	0.49	-	-
110	1536	(E)-Allyl cinnamate	-	0.35	-	-	-	-
111	1545	Elemol	0.30	0.23	0.44	0.19	0.48	-
112	1551	Germacrene B	-	-	-	-	-	0.91
113	1556	(E)-Nerolidol	0.91	0.86	0.55	0.83	0.63	-
114	1560	Santalenone	0.63	-	1.72	0.18	0.47	0.87
115	1565	Maaliol	-	-	-	-	-	2.06
116	1566	(Z)-3-Hexenyl benzoate	0.55	-	-	-	0.38	-
117	1568	Spathulenol	1.65	0.74	7.96	-	-	1.47
118	1569	(E)-α-isomethyl-Ionol acetate	-	0.26	-	0.44	-	-
119	1570	α-Cedrene epoxide	1.14	3.73	6.65	-	-	0.35
120	1571	Dendrolasin	-	0.17	-	-	-	-
121	1572	Caryophyllene oxide	0.97	-	0.21	4.64	6.60	-
122	1573	*cis*-β-Elemenone	-	0.50	-	-	-	-
123	1574	β-Copaen-4-α-ol	0.40	-	0.31	-	0.17	-
124	1576	Globulol	0.32	-	-	-	-	-
125	1578	ar-dihydro-Turmerone	0.54	-	0.56	-	-	0.67
126	1580	Salvial-4(14)-en-1-one	0.16	-	0.33	0.33	0.27	-
127	1584	Carotol	0.52	-	-	0.25	0.35	-
128	1590	Fokienol	-	1.67	0.77	-	-	-
129	1591	β-*trans*-Elemenone	0.32	-	-	0.22	0.43	1.27
130	1595	Khusimone	1.91	0.55	1.28	1.36	1.35	3.22
131	1596	Geranyl isovalerate	0.75	-	-	0.57	0.89	0.38
132	1601	(Z)-Sesquilavandulol	-	-	-	-	0.18	-
133	1603	β-Atlantol	0.57	0.48	0.92	0.23	0.16	1.71
134	1609	Humulene epoxide II	0.76	-	0.71	0.42	0.46	-
135	1612	Isolongifolan-7-α-ol	1.21	0.19	-	-	-	0.39
136	1615	1,10-di-epi-Cubenol	-	1.43	0.86	1.04	1.41	0.17
137	1620	10-epi-γ-Eudesmol	0.73	-	1.45	0.49	0.45	0.64
138	1622	Silphiperfol-6-en-5-one	0.63	0.66	0.85	-	-	0.71
139	1625	α-Acorenol	0.35	-	-	-	-	-
140	1627	Camphoric acid	0.64	-	-	0.78	0.99	-
141	1629	Gossonorol	0.19	-	0.57	-	-	-
142	1633	α-epi-Cadinol	1.12	2.58	2.37	1.54	3.12	1.54
143	1634	α-epi-Muurolol	0.94	-	0.63	-	-	-
144	1640	Cubenol	0.14	-	-	-	0.15	-
145	1641	β-Eudesmol	2.08	0.40	1.21	0.32	1.60	0.58
146	1644	α-Cadinol	2.73	1.27	3.13	0.85	1.62	1.50
147	1644	Citronellyl angelate	-	-	0.82	-	-	-
148	1652	5-Hydroxy-isobornyl isobutanoate	0.42	-	0.28	-	-	-
149	1657	Selin-11-en-4-α-ol	0.19	-	0.93	-	0.23	-
150	1661	(E)-Bisabol-11-ol	1.01	0.62	-	0.63	1.67	0.69
151	1670	14-hydroxy-9-epi-(E)-Caryophyllene	1.53	0.75	1.26	1.20	0.87	1.23
152	1673	5-iso-Cedranol	-	-	-	-	-	0.24
153	1674	Eudesma-4(15),7-dien-1β-ol	1.39	0.28	1.45	0.33	0.36	0.59
154	1678	Shyobunol	0.43	0.22	-	0.35	0.19	0.30
155	1695	Eudesm-7(11)-en-4-ol	0.47	-	-	-	-	0.30
156	1698	n-Heptadecane	-	-	-	-	-	0.23
157	1706	Sesquicineol-2-one	0.50	-	0.33	0.42	0.37	-
158	1734	iso-Longifolol	0.46	-	-	-	-	-
159	1738	(E)-Pseudoisoeugenyl iso butyrate	0.71	-	0.33	-	-	0.40
160	1745	β-Acoradienol	-	-	-	0.12	-	-
161	1752	γ-Costol	-	-	-	-	0.14	-
162	1762	14-oxy-α-Muurolene	0.69	-	0.69	-	0.35	-
163	1832	Cyclopentadecanolide	-	0.14	0.68	-	0.37	-
164	1854	*cis*-Thujopsenic acid	-	-	-	-	-	0.16
165	1855	(Z,Z)-Farnesyl acetone	-	-	-	0.12	0.30	-
Monoterpene hydrocarbons (MH)			7.54	13.15	2.46	20.53	5.68	6.01
Oxygenated monoterpenes (OM)			16.31	33.57	16.74	27.68	10.69	6.02
Sesquiterpene hydrocarbons (SH)			35.43	26.61	28.66	29.04	53.69	59.38
Oxygenated sesquiterpenes (OS)			30.82	18.17	40.36	17.12	27.17	23.23
Aliphatic compounds (AC)			1.64	1.96	2.69	0.26	0.27	2.12
Carboxylic acids and esters (CE)			2.20	1.27	2.30	0.27	-	1.47
Others			1.27	3.01	2.80	2.03	2.19	0.86
Total identified			95.21	97.74	96.83	97.36	99.69	99.09

**Table 2:** Antioxidant IC_50_ values (determined by DPPH and ABTS assay methods) of the HD-EOs obtained from aerial parts of *A. palestina* subjected to different drying methods.

HDEO	IC_50_ (μg/mL)	
	DPPH	ABTS
FR	(1.00 ± 0.03) × 10^−2^	(8.61 ± 0.02) × 10^−2^
ShD	(6.92 ± 0.02) × 0^−2^	(4.81± 0.01) × 10^−2^
SD	(1.31 ± 0.03) × 10^−2^	(1.34 ± 0.01) × 10^−2^
O40D	(1.66 ± 0.06) × 10^−2^	(1.91 ± 0.01) × 10^−2^
O60D	(2.18 ± 0.02) × 10^−2^	(3.08 ± 0.01) × 10^−2^
MWD	(2.84 ± 0.02) × 10^−2^	(5.12 ± 0.04) × 10^−2^
Ascorbic acid	(2.42 ± 3.21) × 10^−3^	(1.92 ± 2.52) × 10^−3^
α-tocopherol	(2.19 ± 9.45) × 10^−3^	(2.07 ± 3.28) × 10^−3^

**Table 3:** TPC, TFC, and antioxidant IC_50_ values (determined by DPPH and ABTS assay methods) of the APM extracts obtained from aerial parts of *A. palestina* subjected to different drying methods.

Drying Method Used to Prepare the APM Extract	TPC (mg GA/g DE)	TFC (mg Q/g DE)	IC_50_ (μg/mL)	
			DPPH	ABTS
ShD	98.04 ± 0.14	289.84 ± 2.38	(4.63 ± 0.06) × 10^−2^	(4.46 ± 0.05) × 10^−2^
SD	105.37 ± 0.19	305.16 ± 3.93	(4.42 ± 0.02) × 10^−2^	(3.87 ± 0.02) × 10^−2^
O40D	82.98 ± 0.62	240.49 ±1.56	(10.67 ± 0.04) × 10^−2^	(7.47 ± 0.03) × 10^−2^
O60D	43.49 ± 0.57	52.94 ± 0.90	(17.51 ± 1.72) × 10^−2^	(23.99 ± 1.62) × 10^−2^
MWD	77.45 ± 0.57	179.70 ± 1.56	(13.45 ± 0.24) × 10^−2^	(7.64 ± 0.09) × 10^−2^
Ascorbic acid	-	-	(1.80 ± 0.06) × 10^−3^	(1.90 ± 0.06) × 10^−3^
α-tocopherol	-	-	(2.30 ± 0.04) × 10^−3^	(1.80 ± 0.01) × 10^−3^

GA: Gallic Acid; Q: Quercetin; DE: Dry extract.

**Table 4:** Correlation matrix of the variables for the APM extracts of *A. palestina* (*p* value > 0.05).

	TPC	TFC	DPPH	ABTS
TPC	1	0.99027	−0.95541	−0.95987
TFC	0.99027	1	−0.95703	−0.94990
DPPH	0.95541	−0.95703	1	0.84878
ABTS	−0.95987	−0.94990	0.84878	1

**Table 5:** Major compounds identified in the LC-MS chromatograms of the different APM extracts obtained from *A. palaestina* subjected to different drying methods.

No.	R_t_	M/Z	M.WT	Mode of Ionization	Classification	Formula	Compound	ShD	SD	O40D	O60D	MWD
1	0.97	117.0194	118.0267	[M − H]^−^	Organic acid	C_4_H_6_O_4_	Succinic acid	+	+	+	+	-
2	1.04	171.0272	170.0199	[M + H]^+^	Phenolic acid	C_7_H_6_O_5_	Gallic Acid	-	+	+	-	+
3	1.18	125.0245	126.0318	[M − H]^−^	Other	C_6_H_6_O_3_	4-Hydroxy-6-Methylpyran-2-one	+	+	+	-	+
4	1.19	289.092	288.0847	[M + H]^+^	Other	C_12_H_16_O_8_	Phlorin	+	-	-	-	+
5	2.53	139.0393	138.032	[M + H]^+^	Phenolic acid	C_7_H_6_O_3_	4-Hydroxybenzoic acid	+	+	+	-	+
6	2.89	191.0559	192.0632	[M − H]^−^	Organic acid	C_7_H_12_O_6_	Quinic acid	+	+	+	+	+
7	2.90	353.0875	354.0948	[M − H]^−^	phenolic acid	C_16_H_18_O_9_	Chlorogenic acid	+	+	+	-	+
8	2.92	163.0392	162.0319	[M + H]^+^	Organic acid	C6H_10_O_3_S	2-Oxo-5-methylthiopentanoic acid	-	-	-	-	+
9	3.22	179.0348	180.0421	[M − H]^−^	Phenolic acid	C_9_H_8_O_4_	Caffeic Acid	+	+	-	+	-
10	3.30	307.0784	306.0712	[M + H]^+^	Flavonoid	C_15_H_14_O_7_	2,3-*trans*-3,4-*trans*-Leucocyanidin	-	+	-	-	+
11	3.74	227.1276	226.1203	[M + H]^+^	Organic acid	C_12_H_18_O_4_	12-hydroxyjasmonic acid	-	-	-	-	+
12	4.61	611.163	610.1558	[M + H]^+^	Flavonoid	C_27_H_30_O_16_	Luteolin-7,3′-di-O-glucoside	-	+	-	-	-
13	4.71	163.0392	162.032	[M + H]^+^	Other	C_9_H_6_O_3_	Isofaurinone	-	-	-	+	-
14	4.75	479.0825	480.0898	[M − H]^-^	Flavonoid	C_21_H_20_O_13_	Myricetin 3-glucoside	+	+	-	-	+
15	4.81	565.156	564.1487	[M + H]^+^	Flavonoid	C_26_H_28_O_14_	Apiin	-	+	-	-	-
16	4.90	193.0505	192.0432	[M + H]^+^	Other	C_10_H_8_O_4_	Scopoletin	-	+	+	-	-
17	4.94	163.0394	162.0321	[M + H]^+^	Other	C_9_H_6_O_3_	Faurinone	-	-	-	+	-
18	5.29	621.1095	622.1167	[M − H]^−^	Flavonoid	C_27_H_26_O_17_	4′-O-(GlcA(1-2)GlcA) Apigenin	+	+	+	-	-
19	5.30	285.0404	286.0477	[M − H]^−^	Flavonoid	C_15_H_10_O_6_	7,3′,4′,5′-Tetrahydroxyflavone	-	+	-	+	+
20	5.58	465.1041	464.0968	[M + H]^+^	Flavonoid	C_21_H_20_O_12_	Hyperoside	+	+	+	-	+
21	5.59	463.0877	464.095	[M − H]^−^	Flavonoid	C_21_H_20_O_12_	Quercetin 3-O-glucoside	+	+	+	-	-
22	5.87	447.0932	448.1001	[M − H]^−^	Flavonoid	C_21_H_20_O_11_	Luteolin 7-O-glucoside	-	+	+	-	+
23	5.88	509.0937	508.0864	[M + H]^+^	Flavonoid	C_22_H_20_O_14_	Patuletin 3-glucuronide	+	-	-	-	-
24	5.95	493.0982	494.1055	[M − H]^−^	Flavonoid	C_22_H_22_O_13_	Laricitrin 3-galactoside	+	+	+	-	+
25	6.28	499.1246	516.1278	[M + H − H_2_O]^+^	Organic acid	C_25_H_24_O_12_	1,3-Dicaffeoylquinic acid	+	+	+	-	+
26	6.57	193.0506	194.0579	[M − H]^−^	Phenolic acid	C_10_H_10_O_4_	Isoferulic acid	-	-	+	-	-
27	6.60	447.0928	448.1001	[M − H]^−^	Flavonoid	C_21_H_20_O_11_	Kaempferol-3-O-glucoside	+	-	+	-	-
28	6.71	431.0971	432.1044	[M − H]^−^	Flavonoid	C_21_H_20_O_10_	Apigenin-7-O-glucoside	+	+	-	-	-
29	6.81	445.0773	446.0846	[M − H]^−^	Flavonoid	C_21_H_18_O_11_	Genistein	+	+	+	+	+
30	6.88	161.0244	162.0317	[M − H]^−^	Other	C_9_H_6_O_3_	7-hydroxy-Coumarin	+	+	+	+	+
31	6.90	163.0395	162.0322	[M + H]^+^	Other	C_9_H_6_O_3_	4-hydroxy-Coumarin	+	+	+	+	+
32	6.91	515.119	516.1262	[M − H]^−^	Other	C_25_H_24_O_12_	1,5-Dicaffeoylquinic acid	+	+	+	-	+
33	6.94	477.1036	478.1109	[M − H]^−^	Flavonoid	C_22_H_22_O_12_	Isorhamnetin 3-glucoside	-	+	-	-	+
34	7.17	507.1136	508.1209	[M − H]^−^	Flavonoid	C_23_H_24_O_13_	Syringetin 3-glucoside	+	+	-	-	+
35	8.42	301.0353	302.0426	[M − H]^−^	Flavonoid	C_15_H_10_O_7_	Quercetin	-	+	-	-	-
36	8.47	287.0559	286.0486	[M + H]^+^	Flavonoid	C_15_H_10_O_6_	Luteolin	-	+	-	+	+
37	8.75	317.0656	316.0583	[M + H]^+^	Flavonoid	C_16_H_12_O_7_	3-O-methyl Quercetin	-	+	+	-	-
38	9.83	269.0462	270.0534	[M − H]^−^	Flavonoid	C_15_H_10_O_5_	Baicalein	-	-	+	-	-
39	9.94	285.04	286.0473	[M − H]^−^	Flavonoid	C_15_H_10_O_6_	Kaempferol	-	+	-	-	-
40	10.05	299.0556	300.0629	[M − H]^−^	Flavonoid	C_16_H_12_O_6_	Hispidulin	-	-	-	-	+
41	10.32	315.0514	316.0587	[M − H]^−^	Flavonoid	C_16_H_12_O_7_	Isorhamnetin	-	+	+	-	-
42	28.26	279.2322	278.2249	[M + H]^+^	Other	C_18_H_30_O_2_	γ-Linolenic acid	+	-	-	+	+
43	28.85	281.2472	280.2399	[M + H]^+^	Other	C_18_H_32_O_2_	(9Z,12Z)-Linoleic acid	-	-	+	-	+

(+): detected; (-): not detected.

## Data Availability

The data presented in this study are available upon request from the corresponding author.

## Supplementary Materials

The following supporting information can be downloaded at: https://www.mdpi.com/article/10.3390/plants12223914/s1, Figure S1: Representative gas chromatograms for oils extracted from *A. palaestina* obtained by hydro-distillation at different drying methods in comparison to the fresh sample. Figure S2: Antioxidant activity (DPPH and ABTS) of the essential oils (EO) from *A. Palestina* obtained by hydro-distillation at different drying methods in comparison to the fresh sample. Figure S3: Antioxidant activity (DPPH and ABTS) of the methanol extract from *A. palestina* (APM) obtained by different drying methods. Figure S4: LC-MS chromatograms (positive and negative modes) of methanol extract from *A. palestina* (APM) obtained at different drying methods. Figure S5: standards. Figure S6. Distribution of TPC and TFC among the different APM extracts obtained using different drying methods. Figure S7. Distribution of DPPH and ABTS antioxidant activities among the different APM extracts obtained using different drying methods.
