# Supplementary material for: Unveiling the Impact of Drying Methods on Phytochemical Composition and Antioxidant Activity of Anthemis palestina
Source: Plants (Basel). 2023 Nov 20;12(22):3914. doi: 10.3390/plants12223914 (PMC10674521; doi:10.3390/plants12223914)
Supplement: Supplementary file 1 [file plants-12-03914-s001.zip › plants-2662932-supplementary.pdf]

Supplymentary

Unveiling the Impact of Drying Methods on Phytochemical Composition and Antioxidant Activity of *Anthemis palestina*

Materials and Methods

Determination of the Total Flavonoids content (TFC):

In a separate volumetric flask (10 mL), an aliquot (1 mL) of extracts with a concentration of 1 mg/mL was obtained, then an amount of 4 mL of distilled water was added, followed by 0.3 mL of sodium nitrite (5% $NaNO_2$, w/v) and allowed to stand for 5 minutes. After that, an amount of 0.3 mL of aluminium chloride (10% $AlCl_3$) was added and incubated for 6 minutes, followed by 2 mL of sodium hydroxide (NaOH, 1 M) and distilled water to make up a solution of 10 ml. Absorbance was measured at 510 nm after 15 minutes. As a control, methanol was employed.

Determination of Total Phenolic Content (TPC):

2.5 mL of Folin-Ciocalteu reagent (2N) (tenfold diluted) and 2 mL of Na_2CO_3 (75 g/L) were added to 0.5 mL of extract. After 15 minutes at room temperature, the absorbance of the mixture was measured with a (UV-Vis) spectrophotometer at 765 nm in comparison to a blank methanol solution. The absorbance was measured at various concentrations (4-20 mg/mL) using gallic acid as a calibration curve standard. All measurements were performed in triplicate. The total phenolic content is expressed as milligrams per gram of gallic acid equivalent.

Antioxidant activity of HDEOs and methanolic extracts

DPPH$^•$ Free Radical Scavenging Activity:

A 0.1mM DPPH• solution was prepared in methanol, and 2 mL of this solution was added to 2 mL of methanol extract solution at various concentrations (0.005, 0.01, 0.05, 0.1, 0.5, and 1.0) mg/mL , and 1 mL of DPPH$^•$ solution was added to 3 mL of essential oil solution in methanol at different concentrations (4.0×10^{-4}, 2×10^{-3}, 2.80×10^{-3}, 4×10^{-3}, 8×10^{-3}) and the absorbance was sample measured at 517

nm by using UV-Vis spectrophotometer. For 30 minutes, these solutions were incubated in the dark. The absorbance was then measured at 517 nm against blank samples lacking scavenger, and the experiment was repeated three times. A standard curve was created using various DPPH• concentrations.

When exposed to radical scavengers, [DPPH•] decreases significantly. The IC50 value was introduced as a parameter for interpreting the results of the DPPH method. This is defined as the substrate concentration that results in a 50% loss of DPPH activity. The linear regression method was used to calculate IC_{50} values from plots of the percent of antiradical activity against the concentration of the tested compounds.

2.7.2 $ABTS^{\bullet +}$ Radical Scavenging Assay

The stock solutions contain ABTS solution (7 mM) and potassium persulphate solution (2.4 mM) ($K_2S_2O_8$). The working solution was then created by combining the two stock solutions in equal parts and allowing them to react in the dark for 16 hours. This solution was diluted with distilled water before use to obtain an absorbance of 0.75 0.02 at 734 nm using a spectrophotometer. 3 mL of $ABTS^{\bullet +}$ solution was added to 2 mL of extracts at different concentrations (0.005, 0.01, 0.05, 0.1 and 0.5) mg/mL, and 1 mL of ABTS•+ solution was added to 3 mL of essential oil solution in methanol at different concentrations (4.0×10^{-4}, 2×10^{-3}, 2.80×10^{-3}, 4×10^{-3} and 8×10^{-3}) and the absorbance was sample measured at 734 nm by using UV-Vis spectrophotometer. Ascorbic acid and α- tocopherol were used as a positive control for the same concentrations of the crude extracts and essential oils. After 5 minutes of mixing, the absorbance was measured at 734 nm with a spectrophotometer, and the percentage of radical scavenging was calculated for each concentration relative to a blank containing no scavenger.

The IC_{50} values were calculated using the linear regression method of plotting the percent of antiradical activity against the concentration of the tested compounds.

3.8. LC-MS analysis of phytochemicals

This instrument was operated using the Ion Source Apollo II ion Funnel electrospray source. The capillary voltage was 2500 V, the nebulizer gas was 2.0 bar, the dry gas (nitrogen) flow was 8 L/min and the dry temperature was 200 ºC. The mass accuracy was < 1 ppm; the mass resolution was 50000 FSR (Full Sensitivity Resolution) and the TOF repetition rate was up to 20 kHz. using Elute UHPLC coupled to a Bruker impact II QTOFMS. Chromatographic separation was preformed using Bruker solo 2.0_C-18 UHPLC column (100 mm x 2.1 mm x 2.0 µm) at a flow rate of 0.51 mL / min and a column temperature of 40C. Solvents: (A) water with 0.05% formic acid and (B) acetonitrile Gradient: 0 – 27 min linear gradient from 5% - 80% B; 27 – 29 min 95% B; 29.1 min 5% B, total analysis time was 35 min on postive and 35 min on negative mode injection volume 3 ul.

Sample preparation: Unknown sample have been dissolved with 100µl DMSO and 900µl MeOH, centrifuge the sample then filter the sample before injection, we Take 1.0 ml and transfer to autosampler and Inject 3.0 ul. (All standard used for identification of ms/z and the retention time).

Standards Used

No	Analyte Name
1	3-Oxocostusic acid
2	4-Hydroxybenzoic acid
3	4-Hydroxy-Coumarin
4	4-OH-Coumarinic acid
5	5,6,4'-Trihydroxy-7,3'-dimethoxyflavone
6	7-Hydroxy-Coumarin
7	Adenosine
8	Aesculetin
9	Ajugoside
10	ALPHA AMYRIN

11	Apigenin
12	Benzoic acid
13	Beta-amyrin
14	Caffeic acid phenethyl ester (cape)
15	Caffeic Acid
16	Caffeine
17	Carnosic acid
18	Catechin
19	Catechol
20	Chlorogenic acid
21	chrysin
22	Cinnamic acid
23	Colchicine
24	Crotonoyl Cosmosiin
25	Ellagic acid
26	Epicatechin
27	Ferulic acid
28	Galangin
29	Galangustin
30	Gallic acid
31	Gallic acid-ethyl
32	Hesperidin
33	Hispidulin
34	Hyperoside
35	Isoorientin
36	Kaempferol
37	Kumatakenin
38	Ladanetin
39	Lupeol
40	Luteolin

41	Luteolin 7-O-glucoside
42	Myricetin
43	Naringenin
44	Naringin
45	p-Coumaric acid
46	Pivalic Acid
47	Procayadnin
48	Quercetin
49	Quercetin 3,3'-dimethyl ether
50	Resveratrol
51	Rosmarinic Acid
52	Rutin
53	Salvianolic acid B
54	Succinic acid
55	Syringic acid
56	Taraxasterol
57	Vanillic acid
58	Vanillin
59	Vitexin

Figures S1-S5

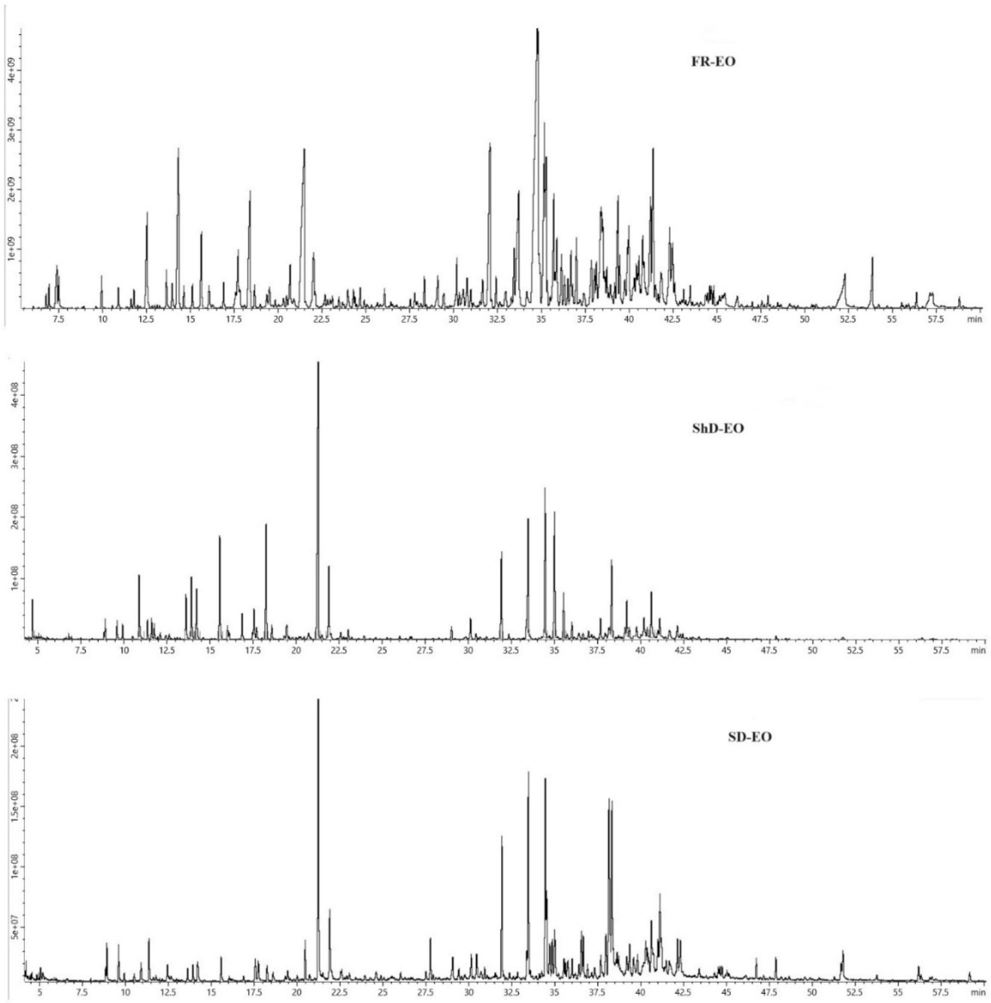

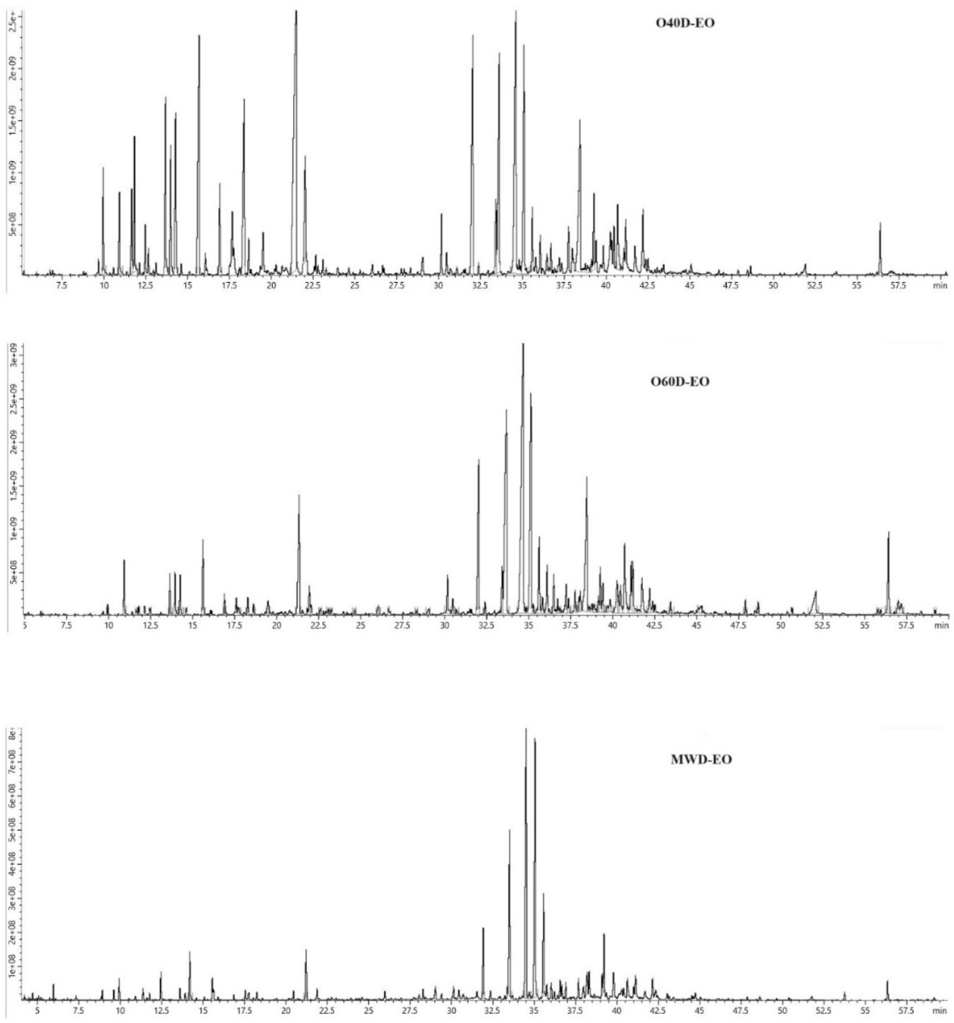

Figure S1: Representative gas chromatograms for oils extracted from *A. palaestina* obtained by hydro-distillation at different drying methods in comparison to the fresh sample.

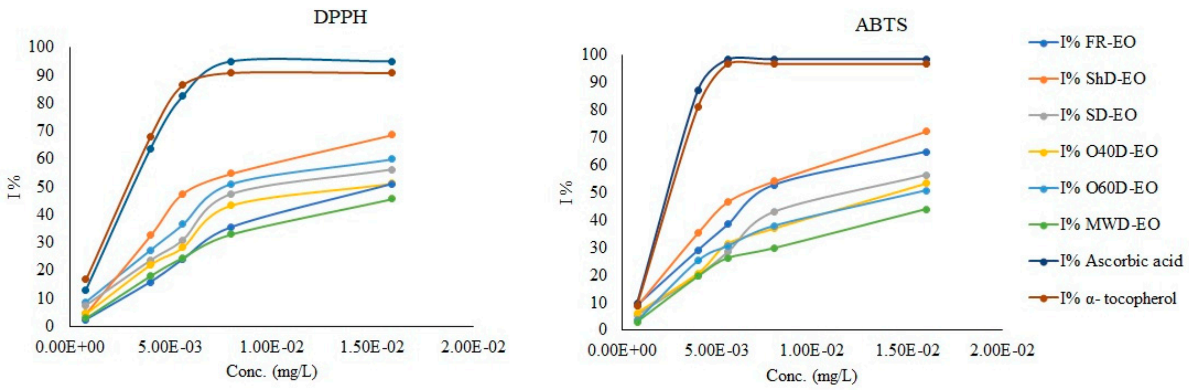

Figure S2: Antioxidant activity (DPPH and ABTS) of the essential oils (EO) from *A. Palestina* obtained by hydro-distillation at different drying methods in comparison to the fresh sample.

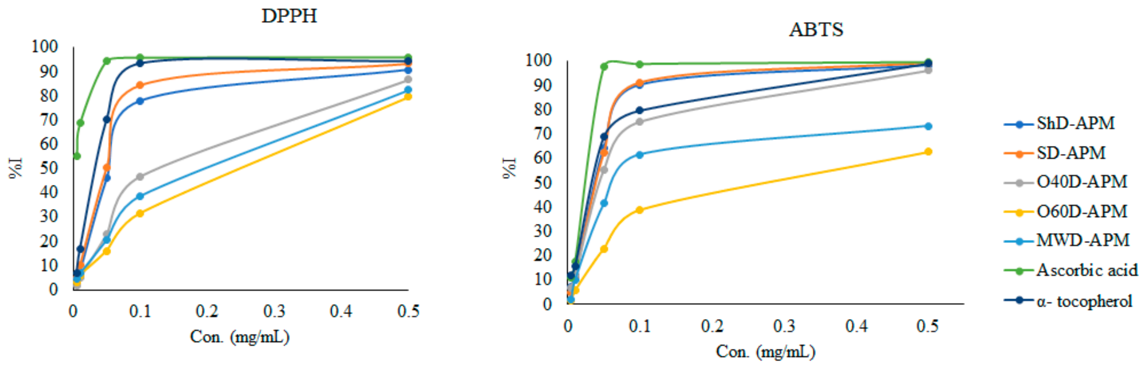

Figure S3: Antioxidant activity (DPPH and ABTS) of the methanol extract from *A. Palestina* (APM) obtained by different drying methods.

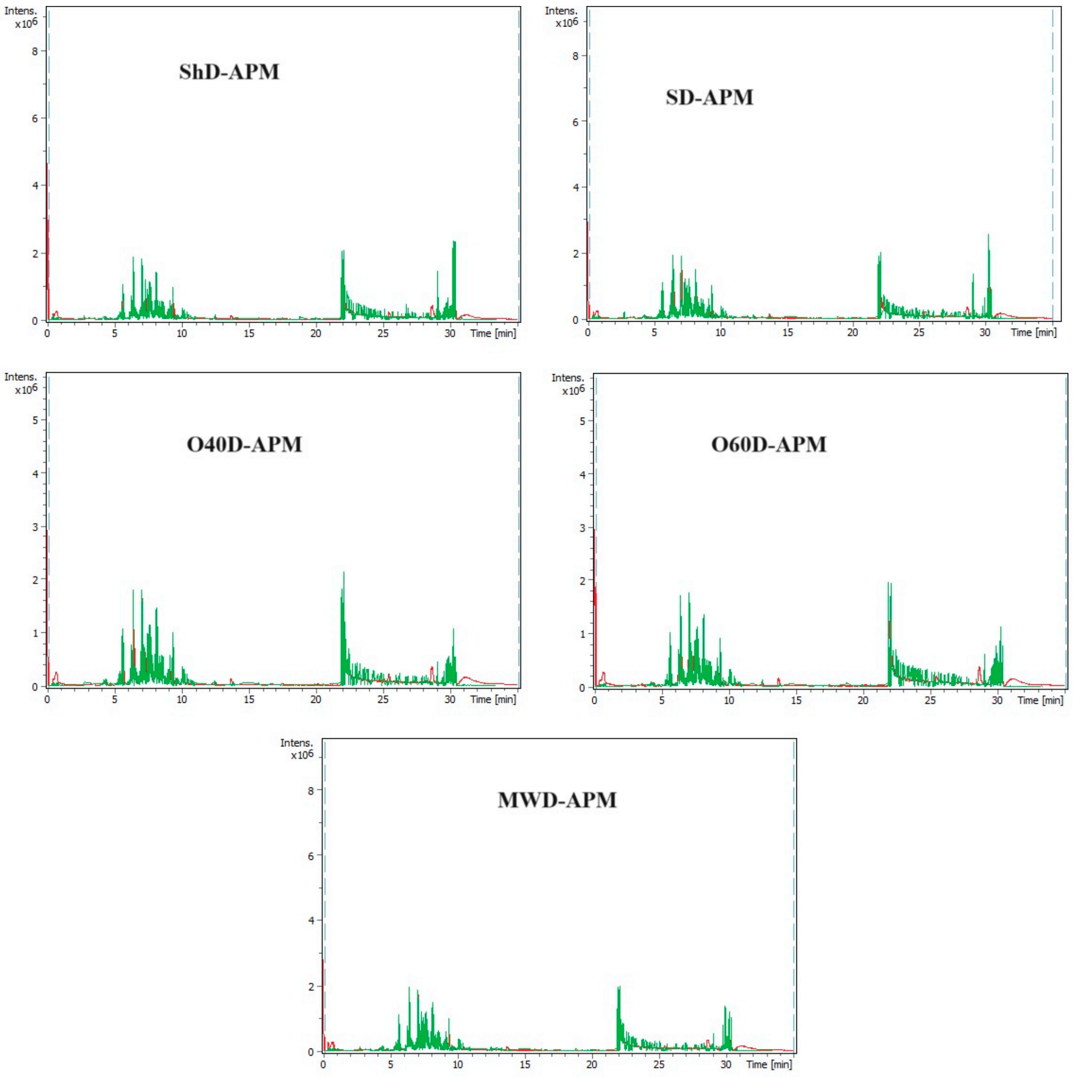

ShD-APM
SD-APM
O40D-APM
O60D-APM
MWD-APM
Intens. x10^6
Time [min]

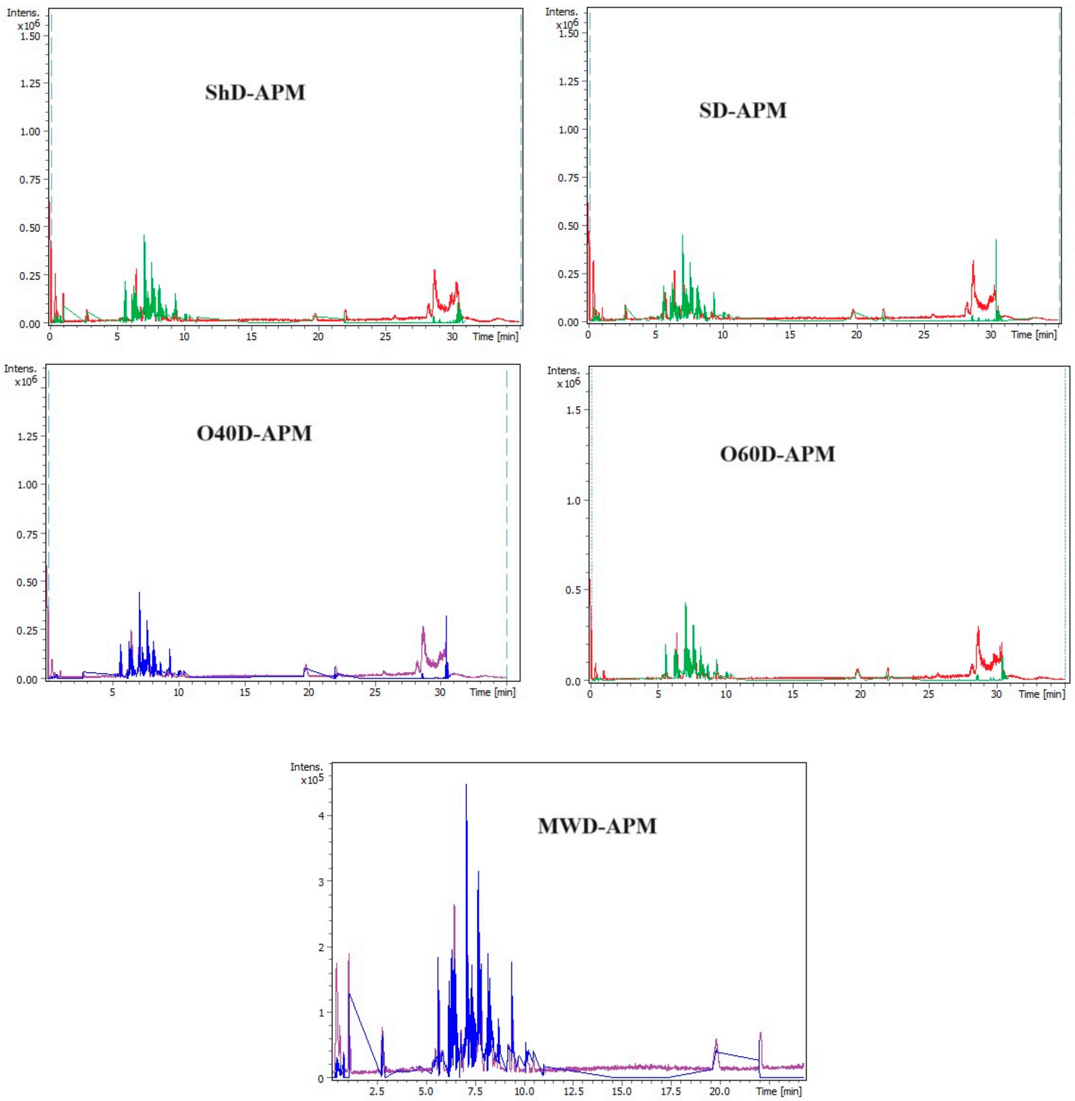

Figure S4: LC-MS chromatograms (positive and negative modes) of methanol extract from *A. palestina* (APM) obtained at different drying methods.

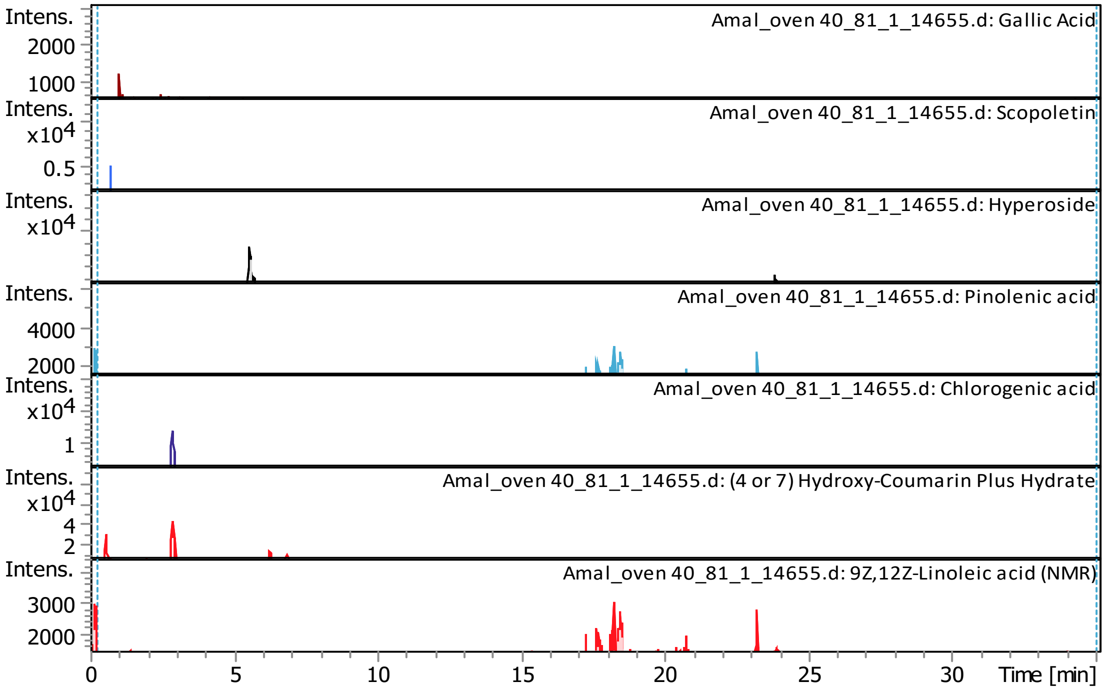

Intens.
2000
1000
Amal_oven 40_81_1_14655.d: Gallic Acid
Intens.
x10^4
0.5
Amal_oven 40_81_1_14655.d: Scopoletin
Intens.
x10^4
Amal_oven 40_81_1_14655.d: Hyperoside
Intens.
4000
2000
Amal_oven 40_81_1_14655.d: Pinolenic acid
Intens.
x10^4
1
Amal_oven 40_81_1_14655.d: Chlorogenic acid
Intens.
x10^4
4
2
Amal_oven 40_81_1_14655.d: (4 or 7) Hydroxy-Coumarin Plus Hydrate
Intens.
3000
2000
Amal_oven 40_81_1_14655.d: 9Z,12Z-Linoleic acid (NMR)
0
5
10
15
20
25
30
Time [min]

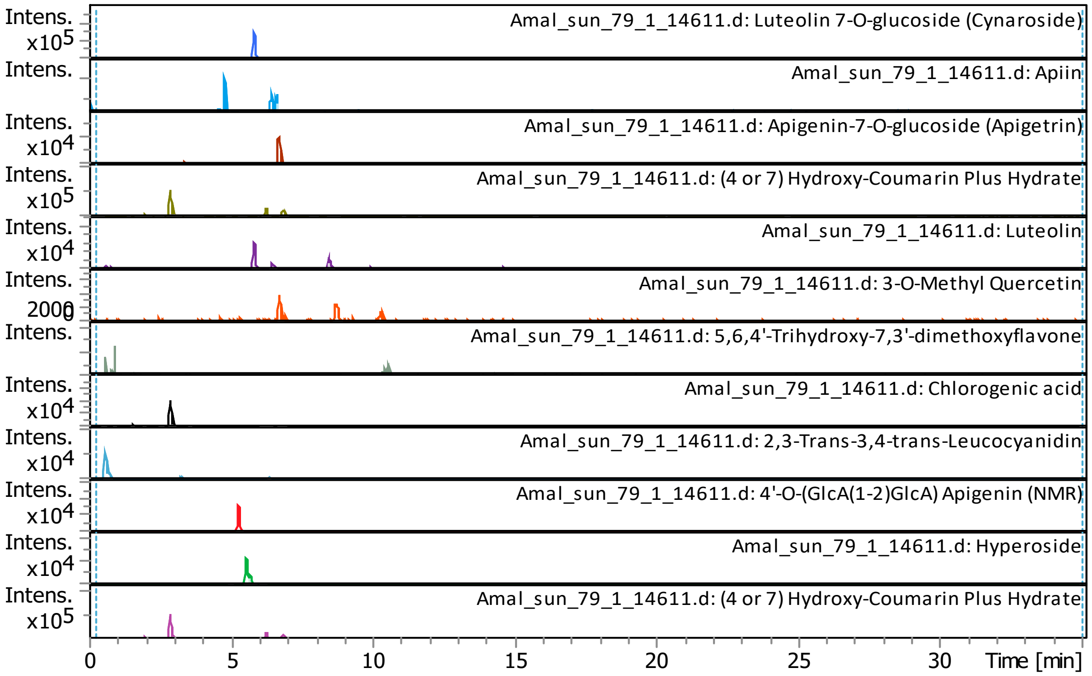
Intens. x10^5
Amal_sun_79_1_14611.d: Luteolin 7-O-glucoside (Cynaroside)
Intens.
Amal_sun_79_1_14611.d: Apiin
Intens. x10^4
Amal_sun_79_1_14611.d: Apigenin-7-O-glucoside (Apigetrin)
Intens. x10^5
Amal_sun_79_1_14611.d: (4 or 7) Hydroxy-Coumarin Plus Hydrate
Intens. x10^4
Amal_sun_79_1_14611.d: Luteolin
Intens. 2000
Amal_sun_79_1_14611.d: 3-O-Methyl Quercetin
Intens.
Amal_sun_79_1_14611.d: 5,6,4'-Trihydroxy-7,3'-dimethoxyflavone
Intens. x10^4
Amal_sun_79_1_14611.d: Chlorogenic acid
Intens. x10^4
Amal_sun_79_1_14611.d: 2,3-Trans-3,4-trans-Leucocyanidin
Intens. x10^4
Amal_sun_79_1_14611.d: 4'-O-(GlcA(1-2)GlcA) Apigenin (NMR)
Intens. x10^4
Amal_sun_79_1_14611.d: Hyperoside
Intens. x10^5
Amal_sun_79_1_14611.d: (4 or 7) Hydroxy-Coumarin Plus Hydrate
0
5
10
15
20
25
30
Time [min]

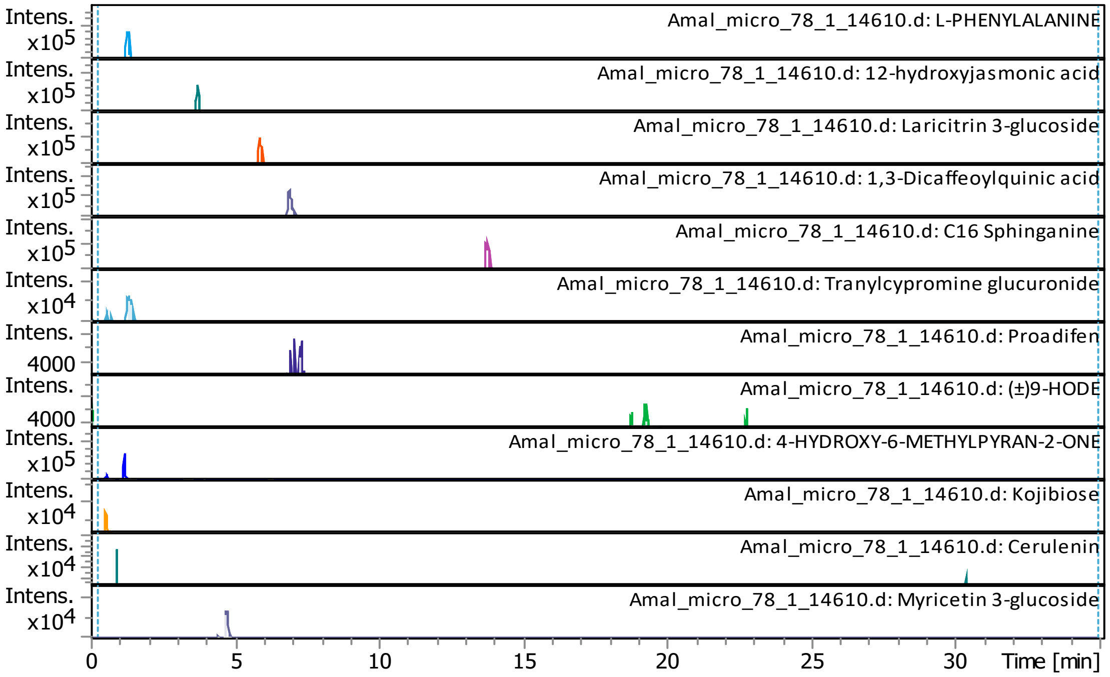
Amal_micro_78_1_14610.d: L-PHENYLALANINE
Amal_micro_78_1_14610.d: 12-hydroxyjasmonic acid
Amal_micro_78_1_14610.d: Laricitrin 3-glucoside
Amal_micro_78_1_14610.d: 1,3-Dicaffeoylquinic acid
Amal_micro_78_1_14610.d: C16 Sphinganine
Amal_micro_78_1_14610.d: Tranylcypromine glucuronide
Amal_micro_78_1_14610.d: Proadifen
Amal_micro_78_1_14610.d: (±)9-HODE
Amal_micro_78_1_14610.d: 4-HYDROXY-6-METHYLPYRAN-2-ONE
Amal_micro_78_1_14610.d: Kojibiose
Amal_micro_78_1_14610.d: Cerulenin
Amal_micro_78_1_14610.d: Myricetin 3-glucoside
Intens. x10^5
Intens. x10^4
Intens. 4000
Time [min]
0
5
10
15
20
25
30

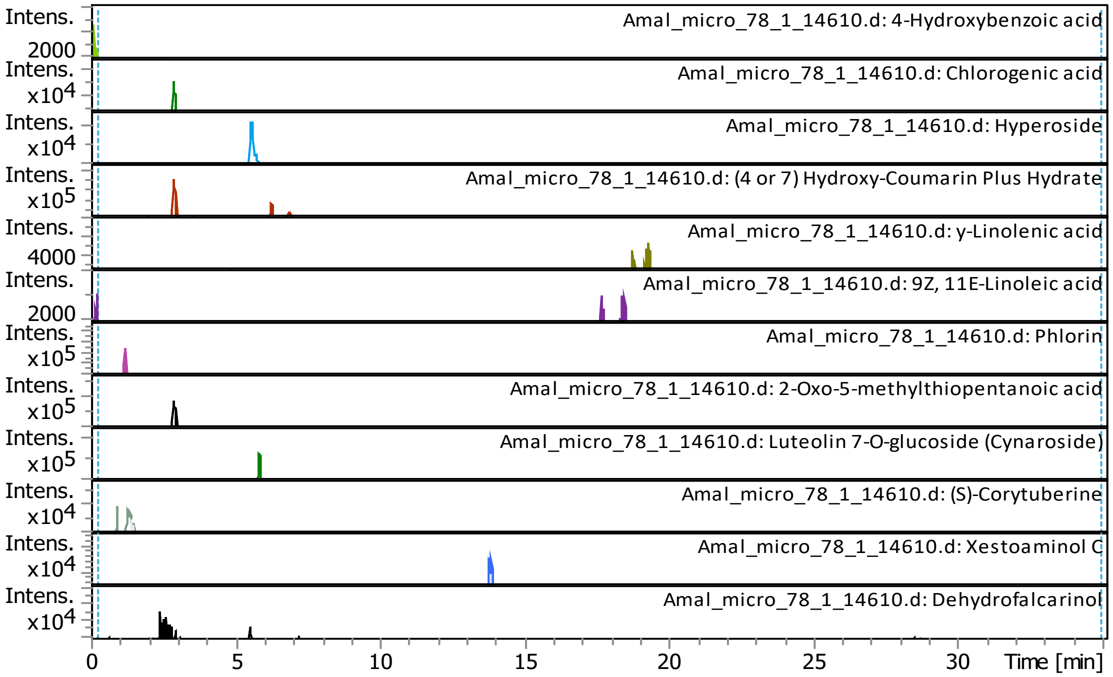

Intens.
2000
Amal_micro_78_1_14610.d: 4-Hydroxybenzoic acid
Intens.
x10^4
Amal_micro_78_1_14610.d: Chlorogenic acid
Intens.
x10^4
Amal_micro_78_1_14610.d: Hyperoside
Intens.
x10^5
Amal_micro_78_1_14610.d: (4 or 7) Hydroxy-Coumarin Plus Hydrate
Intens.
4000
Amal_micro_78_1_14610.d: y-Linolenic acid
Intens.
2000
Amal_micro_78_1_14610.d: 9Z, 11E-Linoleic acid
Intens.
x10^5
Amal_micro_78_1_14610.d: Phlorin
Intens.
x10^5
Amal_micro_78_1_14610.d: 2-Oxo-5-methylthiopentanoic acid
Intens.
x10^5
Amal_micro_78_1_14610.d: Luteolin 7-O-glucoside (Cynaroside)
Intens.
x10^4
Amal_micro_78_1_14610.d: (S)-Corytuberine
Intens.
x10^4
Amal_micro_78_1_14610.d: Xestoaminol C
Intens.
x10^4
Amal_micro_78_1_14610.d: Dehydrofalcarinol
0
5
10
15
20
25
30
Time [min]

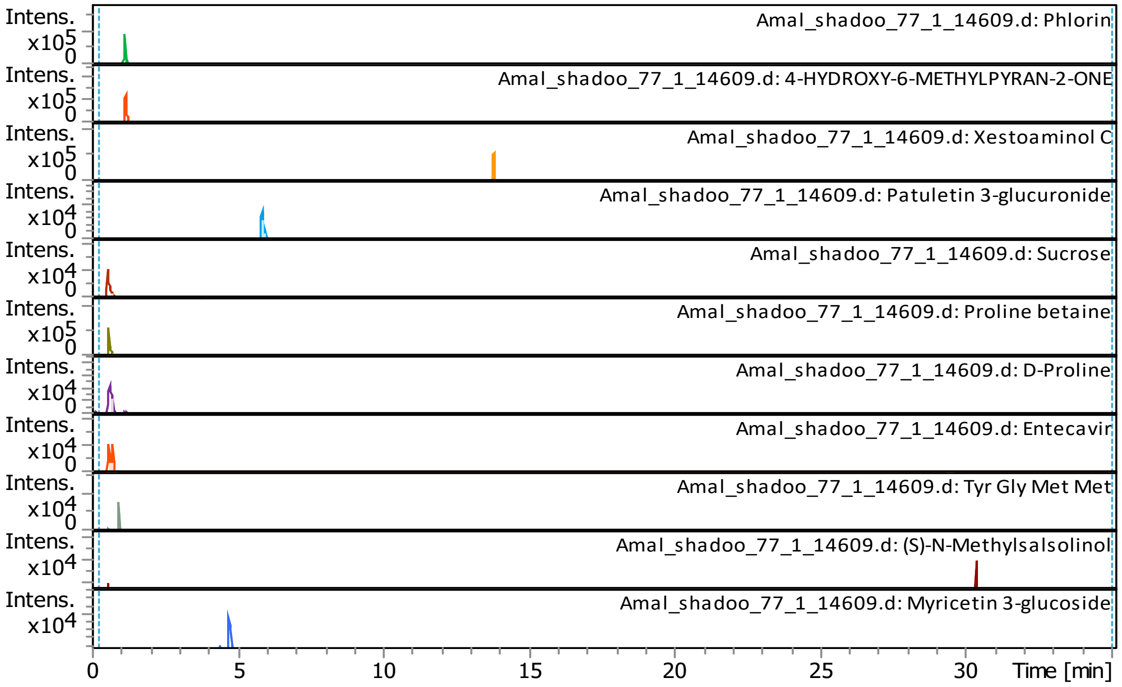

Figure S5: standards

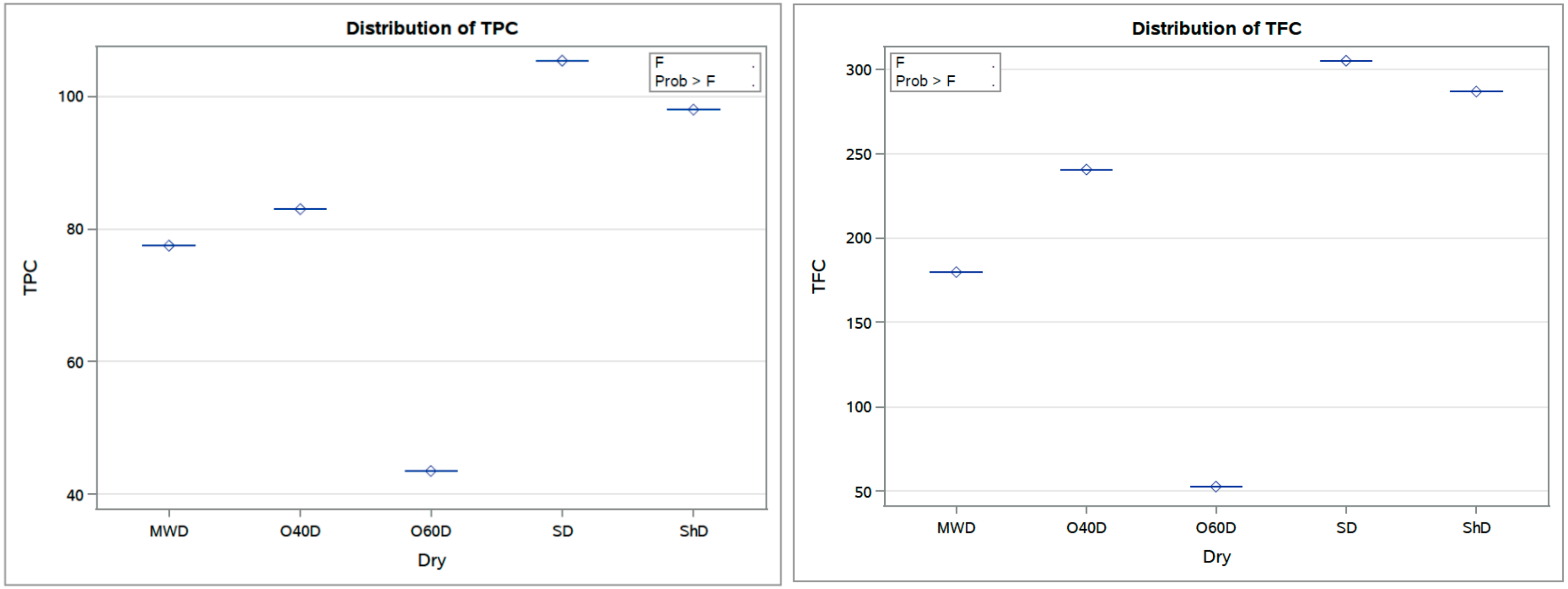

Figure S6. Distribution of TPC and TFC among the different APM extracts obtained using different drying methods.

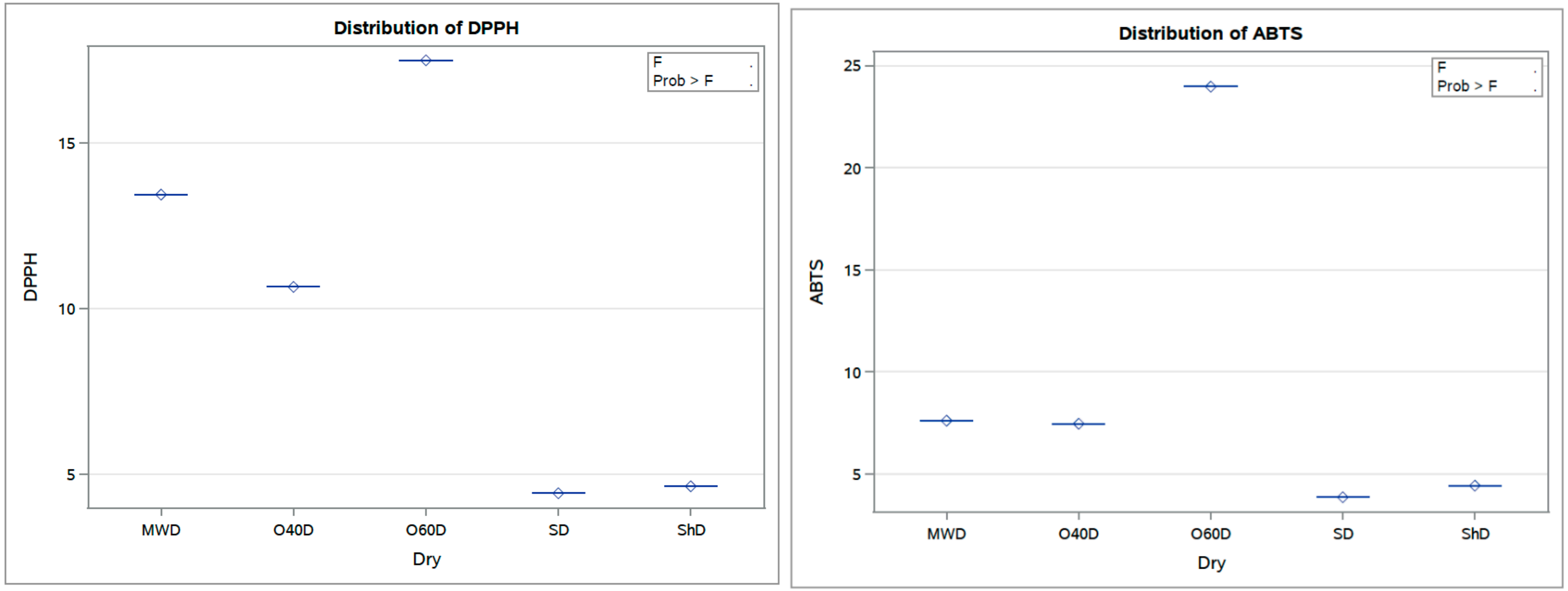

Figure S7. Distribution of DPPH and ABTS antioxidant activities among the different APM extracts obtained using different drying methods.
